# Comparison of the clinical features of human bocavirus and metapneumovirus lower respiratory tract infections in hospitalized children in Suzhou, China

**DOI:** 10.3389/fped.2022.1074484

**Published:** 2023-01-10

**Authors:** Xifeng Tang, Ge Dai, Ting Wang, Huiming Sun, Wujun Jiang, Zhengrong Chen, Yongdong Yan

**Affiliations:** ^1^ Department of Respiratory Medicine, Children's Hospital of Soochow University, Suzhou, China; ^2^ Department of Pediatrics, The First People's Hospital of Yan Cheng, Yancheng, China

**Keywords:** human bocavirus, human metapneumovirus, single infection, comparison, lower respiratory infections

## Abstract

**Objective:**

We compared the clinical data of hospitalized children with lower respiratory tract infections caused by human bocavirus (HBoV) and human metapneumovirus (hMPV).

**Methods:**

In total, 8,430 children admitted to the Department of Respiration, Children's Hospital of Soochow University for lower respiratory tract infections from January 2017 to October 2021 were enrolled. Seven common respiratory viruses, including respiratory syncytial virus, influenza virus A, influenza virus B, parainfluenza virus (PIV) I, PIV II, PIV III, and adenovirus, were detected by direct immunofluorescence assay, whereas human rhinovirus and hMPV were detected by reverse transcription-polymerase chain reaction. *Mycoplasma pneumoniae* (MP) and HBoV were detected by real-time fluorescence quantitative polymerase chain reaction. Bacteria was detected in blood, nasopharyngeal secretion, bronchoalveolar lavage specimen or pleural fluid by culture. In parallel, MP was detected by enzyme-linked immunosorbent assay. In addition, we performed metagenomic testing of alveolar lavage fluid from some of the patients in our study.

**Results:**

The detection rate of HBoV was 6.62% (558/8430), whereas that of hMPV was 2.24% (189/ 8430). The detection rate of HBoV was significantly higher in children aged 1 to <3 years than in other age groups, but there were no significant differences in positivity rates for hMPV by age. Before 2020, the incidence of HBoV infection peaked in summer and autumn, whereas that of hMPV peaked in spring. The epidemiology of both HBoV and hMPV has changed because of the impact of the novel coronavirus. Among the positive cases, the HBoV mixed infection rate was 51.6%, which was similar to that for hMPV mixed infection (44.4%). Comparing clinical characteristics between HBoV and hMPV single infection, the median age of children was 17 months in the HBoV group and 11 months in the hMPV group. In the HBoV single infection group, 31 patients (11.5%) had pulse oxygen saturation of less than 92% on admission, 47 (17.4%) had shortness of breath, and 26 (9.6%) presented with dyspnea. Meanwhile, four patients (3.8%) in the hMPV single infection group had pulse oxygen saturation of less than 92% on admission, eight (7.6%) displayed shortness of breath, and three (2.9%) had dyspnea. The proportion of patients requiring mechanical ventilation and the rate of PICU admission were higher in the HBoV group than in the hMPV group.

**Conclusion:**

The prevalence of HBoV infection is higher than that of hMPV infection in children with lower respiratory tract infection in Suzhou, and HBoV is more likely to cause severe infection than hMPV. Public health interventions for COVID-19 outbreaks have affected the prevalence of HBoV and hMPV.

## 1. Introduction

Lower respiratory infections (LRTIs) in children are among the most common causes of morbidity and mortality globally. Common pathogenic causes of LRTIs include viruses, bacteria, atypical pathogens, and mixed infections, among which viral infections account for at least 60% of cases (1). Human bocavirus (HBoV) and human metapneumovirus (hMPV) are common respiratory viruses in children discovered in 2005 and 2001, respectively, and they are attracting increasing attention.

HBoV, a human microvirus, is an icosahedrally symmetric non-enveloped single-stranded DNA virus. It is classified into four types: HBoV1–HBoV4. HBoV1 is associated with respiratory infections in children, and it is prevalent globally (2). The prevalence of HBoV varies widely based on climatic conditions, geographical differences, and local health levels, and the current prevalence of HBoV respiratory infection is approximately 6.3% (3). Common clinical manifestations of lower respiratory tract infections caused by HBoV include fever, cough, wheezing and dyspnea.

hMPV is a single-negative-stranded RNA virus of the genus *Paramyxoviridae* and family Pneumoviridae (subfamily parapneumovirus) (4). Available studies illustrated that the clinical manifestations of LRTIs caused by hMPV are similar to those of other common respiratory tract infections such as respiratory syncytial virus (RSV) infection without significant specificity.

Although HBoV and hMPV are viral pathogens that cause LRTIs in children with similar clinical manifestations, their respective demographic characteristics, epidemiological patterns, clinical manifestations, laboratory changes, mixed infection types, and disease severity differ. Since the domestic outbreak of the novel coronavirus in late December 2019, the common respiratory virus epidemic has also been affected to varying degrees. This study retrospectively analyzed the clinical data of hospitalized children with LRTIs caused by HBoV or hMPV from January 2017 to October 2021 to compare the demographic and epidemiological characteristics, clinical manifestations, ancillary tests, and treatment and to compare and analyze the epidemiological changes between before and after January 2020 for clinical diagnosis and treatment.

## 2. Materials and methods

### 2.1. Subjects

Children who were admitted to Department of Respiratory Disease at Children's Hospital of Soochow University in Suzhou for LRTIs caused by HBoV and/or hMPV from January 2017 to October 2021 were selected. The included patients ranged in age from 1 month to 12 years. Children were excluded if they had a recent hospitalization (4 weeks before admission), immunodeficiency, a history of a diagnosis of chronic lung disease, congenital airway malformation, or congenital heart disease.

### 2.2. Specimen collection

Nasopharyngeal secretions were collected and analyzed within 24 h of admission to the hospital. The plastic suction hose was inserted through the child's nasal cavity to a depth of 7–8 cm to reach below the pharynx, and 1–2 ml of nasopharyngeal secretions was sucked into a sterile saline tube, shaken, mixed, and then sent for examination within 30 min. We performed bronchoscopy in patients suspected to have airway malformations or those with pulmonary atelectasis. The operator extended the bronchoscope from the nasal cavity through the epiglottis to the trachea and employed physiological saline to irrigate more portions of the airway secretions. The collected alveolar lavage fluid was stored in a sterile collector using a negative pressure suction device and submitted for analysis within 30 min. Seven common respiratory viruses, including RSV, influenza virus A (IVA), influenza virus B (IVB), parainfluenza virus (PIV) I, PIV II, PIV III, and adenovirus (ADV), were detected by a direct immunofluorescence assay, whereas human rhinovirus (HRV) and hMPV were detected by reverse transcription-polymerase chain reaction. *Mycoplasma pneumoniae* (MP) and HBoV were detected by real-time fluorescence quantitative polymerase chain reaction. A bacterial pathogen was defined as detection of *Haemophilus influenza* or *Moraxella catarrhalis*, *Streptococcus pneumoniae*, or *S. pyogenes* in blood, bronchoalveolar lavage specimens or pleural fluid by culture. We quantified the culture of nasopharyngeal secretions and positivity was indicated by a bacterial count of ≥1 × 10^4 ^CFU/ml. MP infection was indicated by a significant increase in MP immunoglobulin G (IgG) levels or the presence of IgM antibodies with MP DNA. Specific IgM and IgG antibodies against MP were detected in the serum samples of patients in the acute and recovery phases of MP, respectively, using a commercial enzyme-linked immunosorbent assay (ELISA) kit (Serion ELISA classic MP IgG/IgM, Institute Virion/Serion, Würzburg, Germany) according to the manufacturer's instructions. The test cutoff was 0.5× mean optical density (OD) of the kit control serum, as indicated in the insert. A positive IgG reaction was defined as a value of >24 RU/ml. A significant increase in the IgG titer was considered a doubling of the OD above the cutoff or a seroconversion in which the primary serum was negative for antibodies and the second serum sample had an OD at least 2-fold the cutoff corresponding to a 3-fold rise in RU/ml. A positive IgM antibody reaction was defined as >1.1 S/CO. In addition, we performed metagenomic testing of alveolar lavage fluid from some of the patients in our study for diagnosis and treatment.

### 2.3. Data collection

Gender, age, duration of illness before admission, cough, fever, wheezing, pulse oxygen saturation on admission, and pulmonary signs on admission were collected. Laboratory findings, such as the white blood cell (WBC) count, neutrophil ratio (N), C-reactive protein (CRP) level, lymphocyte subpopulation distribution, lactate dehydrogenase (LDH) level, nasopharyngeal aspirate or alveolar lavage fluid pathogenic findings, and pulmonary imaging manifestations, were collected from all children. We grouped the subjects by age (28 days to <6 months, 6 months to <1 year, 1 to <3 years, 3 to <5 years, and ≥5 years), season ((spring [March to May], summer [June to August], autumn [September to November], and winter [December to February]), and pathogenic findings (HBoV single infection or hMPV single infection).

### 2.4. Statistical analysis

Data were analyzed using the Statistical Package for the Social Sciences version 26.0 software program (IBM Corporation, Armonk, NY, USA). Data are expressed as percentages, means and standard deviations, or medians and interquartile ranges. Normally distributed continuous variables were compared using a *t*-test, and non-normally distributed variables were analyzed using the Mann–Whitney U-test. Categorical data were analyzed by the chi-squared test or Fisher's exact test. Multiple comparisons were made to identify sources of significant differences, and the results were adjusted using the Bonferroni method. *P* < 0.05 was considered statistically significant.

## 3. Results

### 3.1. Total detection rates of the two viruses

In total, 8,430 children hospitalized for LRTIs from January 2017 to October 2021 were enrolled. Of these patients, 558 (6.62%) were positive for HBoV (469 for nasopharyngeal secretions, 55 for alveolar lavage fluid and 34 for both), and 189 (2.24%) were positive for hMPV (176 for nasopharyngeal secretions, 10 for alveolar lavage fluid and 3 for both), with a statistical difference between the two groups (*χ*^2^ = 190.727, *P* < 0.001).

### 3.2. Demographic characteristics

HBoV-positive patients ranged in age from 28 days to 12 years (median, 20 months). hMPV-positive patients were aged 28 days to 12 years (median, 16 months). The difference in age between these groups was significant (Z = −2.259, *P* = 0.024). The HBoV-positive patients included 349 boys and 209 girls, giving a male-to-female ratio of 1.67:1. The hMPV-positive group included 127 boys and 62 girls, giving a male-to-female ratio of 2.01:1. No statistical difference was found in the gender composition of the two groups (*χ^2^ *= 1.321, *P* = 0.25).

### 3.3. Epidemiological features

#### 3.3.1. Comparison of virus detection rates in different age groups

The HBoV positivity rates among children aged 28 days to <6 months, 6 months to <1 year, 1 to <3 years, 3 to <5 years, and ≥5 years were 2.57%, 6.57%, 12.51%, 6.79%, and 3.01%, respectively (*χ^2^ *= 214.373, *P* < 0.001). Specifically, the positivity rate was significantly higher in children aged 1 to <3 years than in those aged 28 days to <6 months (*χ^2^ *= 157.015, *P* < 0.001), 6 months to <1 year (*χ^2^ *= 28.633, *P* < 0.001), 3 to < 5 years (*χ^2^ *= 32.281, *P* < 0.001), and ≥5 years (*χ^2^ *= 90.445, *P* < 0.001). The hMPV positivity rates among children aged 28 days to <6 months, 6 months to <1 year, 1 to <3 years, 3 to <5 years, and ≥5 years were 2.30%, 2.48%, 2.32%, 2.53%, and 1.47%, respectively, and no differences were detected among the groups (*χ^2^ *= 4.519, *P* = 0.34; Table 1).

**Table 1:** Detection of HBoV and hMPV in different age groups [*n* (%)].

	∼6 m	6 m ∼ 1 y	1 ∼ 3 y	3 ∼ 5 y	≥5 y	*P* values
Total number	2217	1127	2287	1503	1296	—
HBoV	57 (2.57)	74 (6.57)	286 (12.51)	102 (6.79)	39 (3.01)	<0.001
hMPV	51 (2.3)	28 (2.48)	53 (2.32)	38 (2.53)	19 (1.47)	0.34

#### 3.3.2. Comparison of virus detection rates in different gender groups

Of the 558 HBoV-positive cases, 349 were boys, with a positive rate of 6.88%, and 209 were girls, with a positive rate of 6.23%. There was no statistical difference in detection rates by gender (*χ^2^ *= 1.615, *P* = 0.221). In total, 127 of the 189 hMPV-positive patients were boys, with a positive rate of 2.50%, and 62 were girls, with a positive rate of 1.85%. Thus, the detection rate was higher in boys than in girls (*χ^2^ *= 3.96, *P* = 0.047; Table 2).

**Table 2:** Detection of HBoV and hMPV by gender [*n* (%)].

	Boys	Gilrs	*P* values
Total number	5074	3356	—
HBoV	349 (6.88)	209 (6.23)	0.221
hMPV	127 (2.5)	62 (1.85)	0.047

#### 3.3.3. Comparison of virus detection rates in different seasons

**Figure 1:**
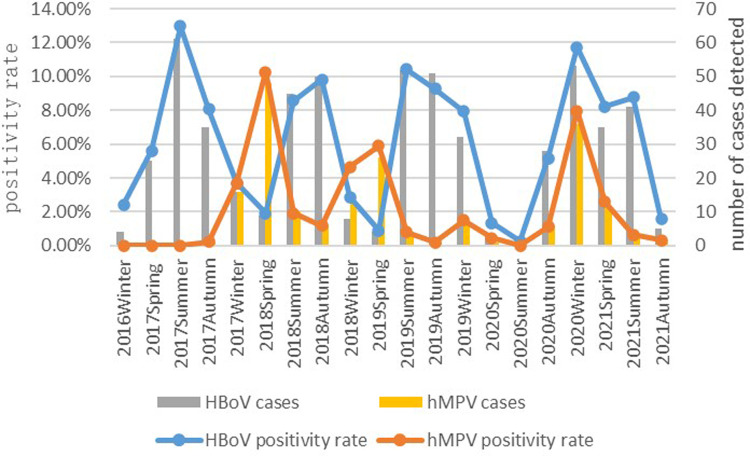
The prevalence of HBoV and hMPV in the past five years by season is shown in Figure 1.

HBoV was detected throughout the year, and epidemic peaks were observed in several seasons over the study period, including summer and fall 2017, summer and fall 2018, summer and fall 2019, winter 2020, and spring and summer 2021. The HBoV detection rates in summer and fall 2020 were significantly lower than those in 2017 (*χ*^2^ = 39.751, *P* < 0.001), 2018 (*χ*^2^ = 29.833, *P* < 0.001), and 2019 (*χ*^2^ = 34.757, *P* < 0.001). There were few positive cases of hMPV in 2017, followed by three epidemic peaks in spring 2018, spring 2019, and winter 2020, and low detection rates were recorded in the remaining seasons (Table 3, Figure 1).

**Table 3:** Detection of HBoV and hMPV in different seasons in Suzhou over the 5-year study period [*n* (%)].

Year	Spring		Summer		Autumn		Winter	
	HBoV	hMPV	HBoV	hMPV	HBoV	hMPV	HBoV	hMPV
2016	—	—	—	—	—	—	4 (2.42)	0 (0.00)
2017	25 (5.61)	0 (0.00)	61 (12.98)	0 (0.00)	35 (8.06)	1 (0.23)	16 (3.71)	16 (3.71)
2018	9 (1.92)	48 (10.26)	45 (8.59)	10 (1.91)	50 (9.82)	6 (1.18)	8 (2.86)	13 (4.64)
2019	4 (0.91)	26 (5.91)	52 (10.42)	4 (0.80)	51 (9.27)	1 (0.18)	32 (7.92)	6 (1.49)
2020	3 (1.34)	1 (0.45)	1 (0.27)	0 (0.00)	28 (5.16)	6 (1.10)	53 (11.73)	36 (7.96)
2021	35 (8.22)	11 (2.58)	41 (8.76)	3 (0.64)	5 (1.56)	1 (0.31)	—	—

### 3.4. Mixed infections

**Figure 2:**
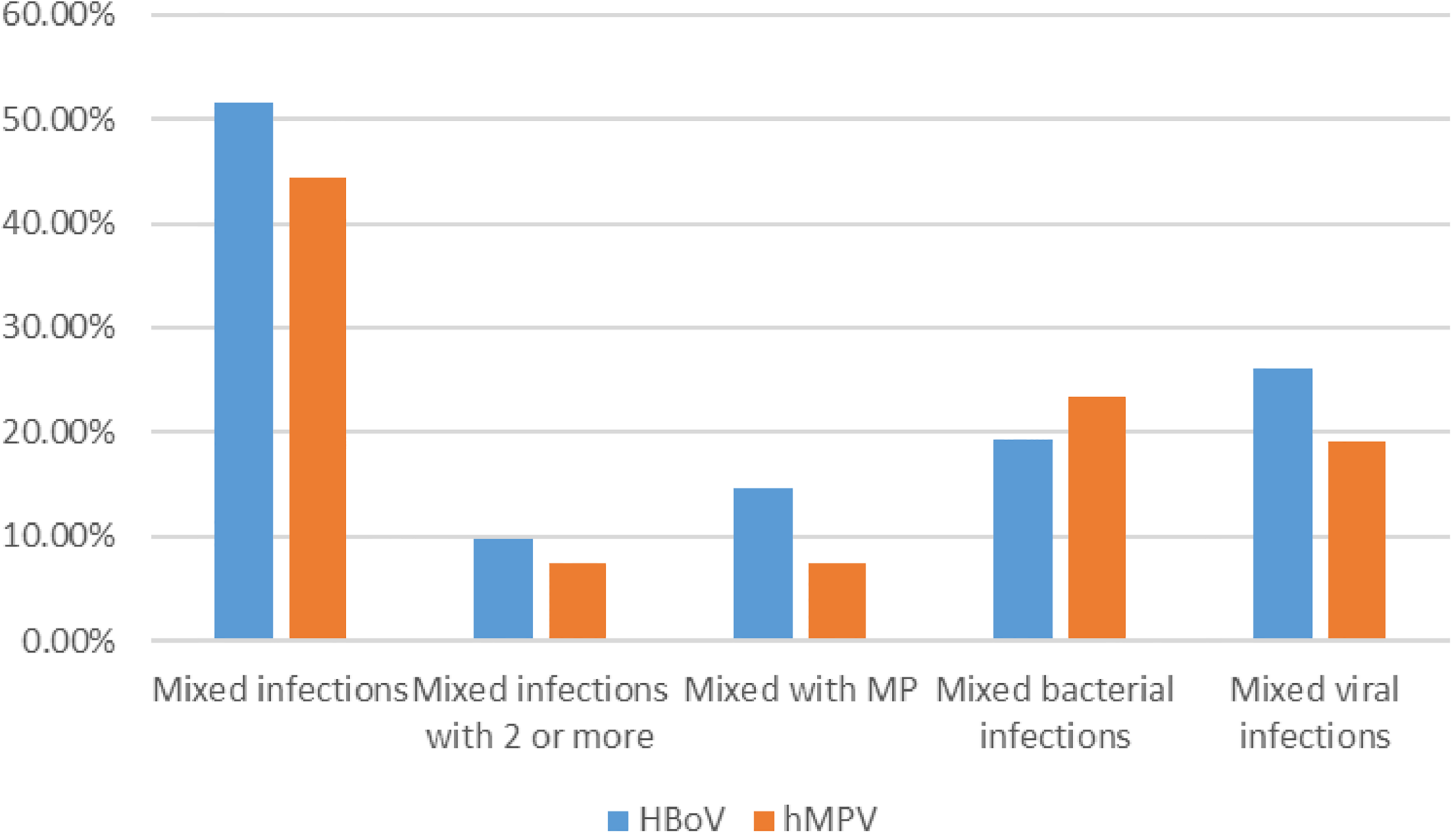
Detection rates of mixed infections in both groups are shown in Figure 2.

Mixed infections were identified in 51.6% (288/558) of the children who were positive for HBoV, 270 of whom (48.39%) had single infections. In total, 234 HBoV-positive patients were infected by one other pathogen, whereas 54 patients were co-infected by two or more additional pathogens. Among the children with mixed infections, 81 (14.52%) had MP infections, and 107 (19.18%) had mixed bacterial infections, including *S. pneumoniae* in 57 children (10.22%), *H. influenzae* in 33 children (5.91%), Catamorium in 14 children (2.51%), and *S. aureus* in 10 children (1.79%). Meanwhile, 146 children (26.16%) had mixed common respiratory viral infections, including HRV in 64 children (11.47%), RSV in 34 children (6.09%), PIV III in 19 children (3.41%), hMPV in 17 children (3.05%), influenza virus (IV) in 5 children (0.90%), and ADV in 6 children (1.08%). hMPV single infections were identified in 105 of 189 patients (55.56%). In total, 84 patients (44.44%) were infected by hMPV together with other pathogens. Co-infection by hMPV and one other pathogen was identified in 70 children (37.04%), whereas co-infected by two or more pathogens was found in 14 children (7.41%). Fourteen patients (7.41%) were infected with hMPV and MP. hMPV and bacteria co-infection was found in 44 patients (23.28%), including *S. pneumoniae* co-infection in 24 patients (12.70%), *H. influenzae* co-infection in 15 patients (7.94%), *S. aureus* co-infection in 2 patients (1.06%), Catamorium co-infection in 2 patients (1.06%), and *H. parainfluenzae* co-infection in 1 patient (0.53%). Thirty-six patients (19.05%) were co-infected by hMPV and other common viruses, including 20 cases (10.58%) of HBoV infection, 2 cases (1.06%) of RSV infection, 7 cases (3.70%) of PIV III infection, 6 cases (3.17%) of HRV infection, and 1 case (0.53%) each of IV and ADV infection. The rates of MP co-infection (*χ^2^ *= 6.427, *P* = 0.011) and mixed common respiratory virus infections (*χ^2^ *= 3.881, *P* = 0.049) were significantly higher in the HBoV group than in the hMPV group. There was no statistical difference between the two groups in terms of the proportion of mixed infections (*χ^2^ *= 2.903, *P* = 0.093), the proportion of co-infection by two or more pathogens (*χ^2^ *= 0.879, *P* = 0.348), and the proportion of bacterial co-infections (*χ^2^ *= 1.475, *P* = 0.225) (Table 4, Figure 2).

**Table 4:** Detection rate of mixed infections in both groups [*n* (%)].

	Mixed infections	Mixed infections with 2 or more	Mixed with MP	Mixed bacterial infections	Mixed viral infections
HBoV	288 (51.61)	54 (9.68)	81 (14.52)	107 (19.18)	146 (26.16)
hMPV	84 (44.44)	14 (7.41)	14 (7.41)	44 (23.28)	36 (19.05)
*χ^2^*	2.903	0.879	6.427	1.475	3.881
*P*	0.093	0.348	0.011	0.225	0.049

Mixed infections: HBoV or hMPV mixed with other pathogens; mixed infections with 2 or more: HBoV or hMPV mixed with 2 or more pathogens; mixed with MP: HBoV or hMPV mixed with MP; mixed bacterial infections: HBoV or hMPV mixed with bacterial; mixed viral infections: HBoV or hMPV mixed with other viruses.

### 3.5. Comparison of the clinical features of hBoV single infections and hMPV single infections

#### 3.5.1. Demographics

The median age of children in the HBoV single infection group was 17 months, vs. 11 months in the hMPV single infection group (Z = −2.73, *P* = 0.007). The proportion of boys in the HBoV single infection group was 67.04%, similar to that in the hMPV single infection group (67.6%).

#### 3.5.2. Clinical presentation

Thirty-one children (11.5%) in the HBoV single infection group had pulse oxygen saturation of less than 92% on admission, 47 (17.4%) had shortness of breath, and 26 (9.6%) presented with dyspnea. Four children (3.8%) in the hMPV single infection group had pulse oxygen saturation of less than 92% on admission, eight children (7.6%) had shortness of breath, and three children (2.9%) displayed dyspnea, and all of these values were lower than those in the HBoV group (*χ^2^ *= 5.258, *P* = 0.028; *χ^2^ *= 5.788, *P* = 0.022; *χ^2^ *= 10.643, *P* = 0.001, respectively). The percentage of children with fever was 68.1% in the HBoV single infection group, vs. 56.2% in the hMPV single infection group (*χ^2^ *= 4.739, *P* = 0.031). There were no statistical differences between the two groups regarding the rates of cough, wheeze, stuffy and runny nose, cyanosis, gastrointestinal symptoms, low breath sounds, stridor, and crackles.

#### 3.5.3. Ancillary examinations

The median WBC count was 9.86 × 10^9^/l and N was 44.95% in the HBoV single infection group, both of which were higher than those of the hMPV single infection group (Z = −2.35, *P* = 0.018; Z = −4.156, *P* = 0.001, respectively). Ninety-nine children (36.7%) in the HBoV single infection group displayed elevated CRP levels, compared with 26 children (24.8%) in the hMPV group (*χ^2^ *= 4.821, *P* = 0.038). There were differences in cellular immunity between the two groups. The CD3−CD16 + CD56 + ratio was lower in the HBoV single infection group than in the hMPV single infection group (Z = −2.584, *P* = 0.011), whereas the CD19 + CD23 + ratio was higher (Z = −2.283, *P* = 0.024). No statistical difference was found between the two groups in terms of imaging performance.

#### 3.5.4. Treatment

Oxygen support was given to 47 patients (17.4%) in the HBoV single infection group and 11 patients (10.5%) in the hMPV single infection group, with no statistical difference between the two groups. In the HBoV single infection group, nine patients (3.3%) required assisted mechanical ventilation, whereas no patients needed assisted mechanical ventilation in the hMPV group. Meanwhile, 26 patients (9.6%) in the HBoV group were admitted to the PICU, compared with one patient (1.0%) in the hMPV group (*χ^2^ *= 8.519, *P* = 0.003).

#### 3.5.5. Length of stay

The median length of stay was 7 days in both groups (Table 5).

**Table 5:** Comparison of the clinical features of HBoV and hMPV single infections [*n* (%)].

	Clinical Information	HBoV single infection	hMPV single infection	*P* values
Demography	Sex, *n* (%) male	181(67.04)	71(67.6)	1
	Age, months	17 (11, 30)	11 (4, 40)	0.007
Clinical presentation	Pulse oxygen below 92%, *n* (%)	31(11.5)	4(3.8)	0.028
	Fever, *n* (%)	184(68.1)	59(56.2)	0.031
	Cough, *n* (%)	260 (96.3)	104 (99.0)	0.193
	Wheeze, *n* (%)	130(48.1)	53(50.5)	0.73
	Stuffy nose rhinorrhea, *n* (%)	114(42.2)	46(43.8)	0.817
	Dyspnea, *n* (%)	26(9.6)	3(2.9)	0.001
	Shortness of breath, *n* (%)	47(17.4)	8(7.6)	0.022
	Cyanosis, *n* (%)	7 (2.6)	2 (1.9)	0.74
	Gastrointestinal symptoms, *n* (%)	61(22.6)	24(22.9)	1
Auscultation	Low breath sounds, *n* (%)	14(5.6)	3(2.9)	0.417
	Stridor, *n* (%)	125 (46.3)	53 (50.5)	0.491
	Cracklesfing, *n* (%)	194(71.9)	81(77.1)	0.363
Ancillary examinations	WBC count, × 10^9^/L	9.86 (7.62, 12.84)	8.8 (6.68, 12.28)	0.018
	Percentage of neutrophils, %	44.95 (29.9, 62.33)	31.3 (21.15, 50.65)	0.001
	PLT count, × 10^9^/ml	337 (268, 420)	321 (250, 440)	0.652
	CRP > 8 mg/L, n (%)	99(36.7)	26(24.8)	0.038
	ALT > 40 U/L, n (%)	19(7.0)	11(10.5)	0.291
	LDH > 382 U/L, n (%)	157(58.1)	57(54.3)	0.561
	CD3+, %	60.9 (54.8, 68.4)	59.9 (53.6, 67.9)	0.716
	CD3 + CD4+, %	34.75 ± 10.46	36.55 ± 10.75	0.189
	CD3 + CD8+, %	21.7 (17.1, 27.9)	20.6 (16.23, 25.35)	0.13
	CD4+/CD8+, %	1.7 (1.2, 2.38)	1.7 (1.2, 2.5)	0.402
	CD3-CD19+, %	26.1 (19.28, 31.95)	23.25 (15.03, 32.2)	0.079
	CD3-CD16 + CD56+, %	8.2 (5.03, 14.2)	10.90 (5.68, 19.4)	0.011
	CD19 + CD23+, %	11.3 (7.43, 16.38)	9.35 (6.05, 14.45)	0.024
	Deepening of lung marking, *n* (%)	46(17.8)	21(20.6)	0.549
	Dotted or small patchy shadows, *n* (%)	183(70.7)	75(73.5)	0.608
	Large lesions or solid shadows, *n* (%)	30(11.6)	6(5.9)	0.12
Treatment	Oxygen support, *n* (%)	47(17.4)	11(10.5)	0.096
	Mechanical ventilation, *n* (%)	9(3.3)	0	—
	Admission to PICU, *n* (%)	26(9.6)	1(1.0)	0.003
	Length of stay, *d*	7 (6, 9)	7 (6, 9)	0.99

Lypmphocyte subset analysis was performed in 212 patients in HBoV group and 83 patients in hMPV group.

## 4. Discussion

HBoV is a common respiratory virus in children. Because of differences in regions, climatic environments, and other factors, the reported detection rate of HBoV varies widely by location. hMPV can cause mild upper respiratory infections, as well as capillary bronchitis and pneumonia. HBoV and hMPV are still under-researched and poorly understood. To provide a reference for clinical diagnosis and management, we analyzed and compared the demographic and epidemiological characteristics, clinical manifestations, and laboratory and imaging changes of LRTIS caused by HBoV and hMPV in Suzhou over a 5-year period.

The detection rate of HBoV among hospitalized children with LRTIs in Suzhou from January 2017 to October 2021 was 6.62%. A previous report recorded a detection rate of 9.38% in Nanjing, China (5). The reported HBoV detection rate in Korea is 12.2% (6). HBoV detection rates vary by region and climate. The median age of the HBoV group in this study was 20 months, suggesting susceptibility to HBoV in children in infancy and early childhood. This is considered to be related to the underdevelopment of the respiratory and immune systems of children in this age group. The detection rate of children aged 1 to <3 years in this study was 12.51%, which was significantly higher than that in the other age groups. In the Suzhou area, HBoV is mainly prevalent in summer and autumn. In Brazil and Croatia, the highest detection rates were recorded in winter (7, 8). Peaks in the HBoV prevalence were observed in summer and autumn in 2017, 2018, and 2019 in this study, whereas HBoV detection rates were significantly lower in summer and autumn 2020 than in previous years, with peaks observed in winter 2020 and spring and summer 2021. This may be related to the government's public interventions to stop the novel coronavirus epidemic, such as prohibiting markets or other forms of gatherings and closing theaters, cafes, and public bathrooms, and such strategies may have also effectively curbed the spread of other pathogens that cause respiratory infections. Previous studies by our research group indicated that public interventions by the government can influence the prevalence of common respiratory viruses (9). We need to continuously monitor the prevalence of respiratory viruses. The possibility of a rebound or even a pandemic of common respiratory viruses in the later stages of the epidemic because of relaxed prevention cannot be dismissed (10).

The results of this study demonstrated that the detection rate of hMPV in Suzhou was 2.24%. The prevalence rate is 4.7% in Africa (11) and 3.6% in Bangkok, Thailand (12). The median age of children infected with hMPV was 16 months, which was significantly lower than that of children infected with HBoV. Unlike HBoV, the hMPV detection rate was significantly higher in boys than in girls (2.5% vs. 1.85%). However, some scholars reported that there is no difference in the detection rate of hMPV in different genders (13). hMPVs are mainly distributed from December to May of the following year in Guangzhou, China (14). Belgium and Japan have reported higher hMPV prevalence in winter and spring (15, 16). In this study, only two patients were positive for hMPV in the entire year of 2017, followed by epidemic peaks in spring 2018 and 2019, whereas HBoV was mainly prevalent in summer and autumn in Suzhou. There are significant differences in the epidemic seasons of the two viruses. Since December 2019, there has been an outbreak of the COVID-19 pandemic in China. The hMPV detection rate in the Suzhou region was only 0.45% in spring 2020, which was significantly lower than that in spring 2018, 2019, and 2021, and another epidemic peak of hMPV was observed in winter 2020. A study found that the prevalence of common respiratory viruses such as IV, RSV, and hMPV fell to historic lows during the COVID-19 pandemic, which was linked to a strict policy of public health interventions in response to the outbreak. Clinicians need to be aware that during an epidemic, common respiratory viruses may not display their typical seasonal cycles (17).

HBoV is often detected together with other pathogens, and some studies found that the rate of mixed infections with HBoV can be as high as 75% (18). In this study, the total mixed HBoV infection rate was 51.61%, and 107 mixed infections involved bacteria (37.15%), with *S. pneumoniae* and *H. influenzae* co-infection being the most common. Mixed MP infections accounted for 28.13% of all mixed infections, and mixed viral infections accounted for 50.69% of mixed infections. Among viral mixed infections, HRV co-infection was most common, followed by RSV and PIV III co-infection. One report found that among 88-HBoV positive cases in Ningxia, China, there were 80 (91.9%) mixed infections, including 76 (95.0%) mixed viral infections, and HRV co-infection was most common, followed by RSV. Twenty-nine patients were infected with hMPV (36.25%) and MP, and 24 (30.0%) had mixed bacterial infections, with *Escherichia coli* and *Klebsiella pneumoniae* being the most common co-pathogens (19). HBoV is the fourth most common respiratory virus in Korea, and the prevalence of mixed viral infection with HBoV is 57.2%, with the most common mixed viruses being HRV, ADV, and hMPV (6). The common mixed pathogens of HBoV vary by region. This may be related to the different epidemiological characteristics of the different respiratory pathogens in each region. From a single pathogen perspective, the high prevalence of with HBoV mixed with MP and HRV in this study may be related to the high prevalence of MP and HRV in summer and autumn in China (20, 21).

The proportion of mixed MP for hMPV and the proportion of mixed virus infections were significantly lower than the findings for HBoV. These findings are associated with the epidemiological differences between the two viruses. Reports from Changsha and Beijing, China indicated that the most common respiratory virus causing co-infection with hMPV is RSV (13, 22). By contrast, hMPV mixed infections in Madrid, Spain most frequently involve ADV and HRV (23). Different regions have different mixed respiratory viruses, which may be related to the climate, environment, and epidemiological characteristics of different respiratory viruses in different regions.

In this study, HBoV single infections were more common in children aged 1 to <3 years, and hMPV infections were more common in children younger than 1 year. The median age of the HBoV single infection group was 17 months, whereas that of the hMPV group was 11 months. In the HBoV single infection group, 11.5% of the children had pulse oxygen saturation of less than 92% at admission, 9.6% had dyspnea, and 17.4% had shortness of breath, and these values were significantly higher than those in the hMPV single infection group. Therefore, we concluded that HBoV is more likely to cause severe infection than hMPV. The pathogenesis of these infections is unclear. The ratio of CD19 + CD23 + cells in the HBoV single infection group was higher than that in the hMPV single infection group, whereas the proportion of CD3−CD16 + CD56 + cells was lower in the HBoV group, suggesting that B cell activation and low NK cell function are possible causes of the observed pathogenesis.

Wheezing is one of the common clinical manifestations in children infected with HBoV or hMPV in our study. A study found that 13.0%–60.7% of children infected with hMPV had recurrent wheezing or a diagnosis of asthma (24). A cohort study also found that early severe HBoV infections were associated with the development of asthma in children aged 5–7 years (25). Both HBoV and hMPV infections can cause cellular immune disorders, and the median CD19 + CD23 + cell count is elevated by these viruses. CD19 and CD23 positivity is a marker of B lymphocyte activation and differentiation, and the number of CD19 + CD23 + cells represent the number of activated B lymphocytes. Upon activation, B lymphocytes induce the production of IgE, which is closely related to the occurrence, development, and attack of allergic diseases including asthma. Some authors believe that CD23 + cells can serve as a therapeutic target for allergy (26). CD19 + CD23 + cells have important predictive value for the prognosis of infantile wheezing. HBoV and hMPV infections have a certain relationship with atopic inflammation, which leads to the occurrence of wheezing. In our follow-up study, we will focus on the impact of these two viruses on the prognosis of children with allergic disease.

In conclusion, the detection rate of HBoV is higher than that of hMPV in children with respiratory tract infections in the Suzhou area. HBoV infections are more common in children aged 1–3 years. The incidence of HBoV usually peaks in summer and autumn, whereas hMPV is more prevalent in spring. The two viruses have similar clinical manifestations, including fever, cough, and wheezing, but HBoV is more likely to cause severe infection than hMPV. Public health interventions for COVID-19 outbreaks have affected the prevalence of both HBoV and hMPV.

## Data Availability

The raw data supporting the conclusions of this article will be made available by the authors, without undue reservation.
